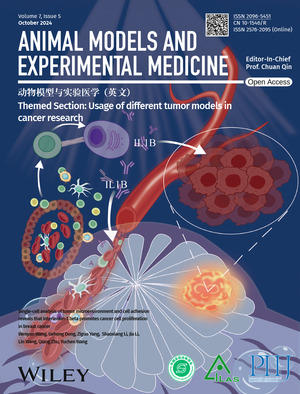# Cover Picture

**DOI:** 10.1002/ame2.12493

**Published:** 2024-11-01

## Abstract

The cover image is based on the article ‘Single‐cell analysis of tumor microenvironment and cell adhesion reveals that interleukin‐1 beta promotes cancer cell proliferation in breast cancer’ (DOI: 10.1002/ame2.12445) reported by Wenyan Wang, Gehong Dong, Ziguo Yang, Shaoxiang Li, Jia Li, Lin Wang, Qiang Zhu, Yuchen Wang. The cover features the body contour of a breast cancer patient, with a tumor located in the upper outer quadrant of the left breast. A magnified view reveals the microscopic structure of breast tumor cells. The tumor tissue is richly vascularized, and below it, the tumor microenvironment is depicted, composed of various cellular clusters. Notably, plasma cells are shown accumulating around triple‐negative breast cancer (TNBC) cells. These plasma cells interact with T cells via intercellular adhesion molecule 2 (ICAM‐2) and the integrin complex aLb2, and promote tumor growth by releasing interleukin‐1 beta (IL1B). This cover illustration highlights the critical role of plasma cells in TNBC and identifies IL1B as a novel prognostic marker for TNBC.